# Physicians’ misperceived cardiovascular risk and therapeutic inertia as determinants of low LDL-cholesterol targets achievement in diabetes

**DOI:** 10.1186/s12933-022-01495-8

**Published:** 2022-04-26

**Authors:** Mario Luca Morieri, Olga Lamacchia, Enzo Manzato, Andrea Giaccari, Angelo Avogardo, Lipid-Lowering-Relevance Study Group, Lucio Amoresano, Stefania Angotti, Laura Bartone, Francesco Caraffa, Antonello Carboni, Stefano Carro, Silvestre Cervone, Alessandra Clerico, Ida Console, Danilo Mario Conti, Sergio D’Addato, Alessandra de Bellis, Francesco de Meo, Alberto di Carlo, Graziano di Cianni, Giuseppe di Giovanni, Sergio di Lembo, Fabrizio Diacono, Mara Dolcino, Giovanni Elia, Paolo Elli, Cristina Fatone, Angelica Galli, Giovanni Galluzzo, Adriana Garzaniti, Renata Ghelardi, Anna Giacchini, Loretta Giunta, Francesco Golia, Franco Gregorio, Dario Ierna, Antonio Lampitella, Antonio Luciano, Ada Maffettone, Raffaele Mancini, Ida Mangone, Linneo Enzo Mantovani, Alberto Marangoni, Giuseppe Marelli, Narciso Marin, Gennaro Marino, Eugenio Mastromatteo, Gaetano Mazziotti, Elisa Me, Giuseppe Memoli, Laura Silvia Maria Menicatti, Simona Moffa, Manuela Moise’, Fabrizio Monaco, Sara Nazzarena Morgante, Francesca Pellicano, Ettore Petraroli, Deamaria Piersanti, Antonino Pipitone, Susanna Puglisi, Maura Rinaldi, Mario Rizzo, Maura Rosco, Giampaolo Scollo, Natalino Simioni, Mariarosaria Squadrone, Giacomo Sturniolo, Anna Tedeschi, Biagio Tizio, Diletta Ugolotti, Livio Valente, Carmela Vinci, Luca Zenoni, Maria Grazia Zenti

**Affiliations:** 1 Diabetes Unit, Department of Medicine, University of Padova, via Giustiniani 2 IT, 35128 Padova, Padua Italy; 2 Department of Medical and Surgical Sciences, University of Foggia, Foggia, Italy; 3 Center for Endocrine and Metabolic Diseases, Department of Surgical and Medical Sciences, Fondazione Policlinico Universitario A. Gemelli IRCCS-and Università Cattolica del Sacro Cuore, Rome , Italy

**Keywords:** Inertia, Cardiovascular risk, Misperceived risk, Statins, Ezetimibe, PCSK9i, Real-world study, Adherence, Primary care physicians, Real-world, Self-reported survey

## Abstract

**Background:**

Greater efforts are needed to overcome the worldwide reported low achievement of LDL-c targets. This survey aimed to dissect whether and how the physician-based evaluation of patients with diabetes is associated with the achievement of LDL-c targets.

**Methods:**

This cross-sectional self-reported survey interviewed physicians working in 67 outpatient services in Italy, collecting records on 2844 patients with diabetes. Each physician reported a median of 47 records (IQR 42–49) and, for each of them, the physician specified its perceived cardiovascular risk, LDL-c targets, and the suggested refinement in lipid-lowering-treatment (LLT). These physician-based evaluations were then compared to recommendations from EAS/EASD guidelines.

**Results:**

Collected records were mostly from patients with type 2 diabetes (94%), at very-high (72%) or high-cardiovascular risk (27%). Physician-based assessments of cardiovascular risk and of LDL-c targets, as compared to guidelines recommendation, were misclassified in 34.7% of the records. The misperceived assessment was significantly higher among females and those on primary prevention and was associated with 67% lower odds of achieving guidelines-recommended LDL-c targets (OR 0.33, p < 0.0001). Peripheral artery disease, target organ damage and LLT-initiated by primary-care-physicians were all factors associated with therapeutic-inertia (i.e., lower than expected probability of receiving high-intensity LLT). Physician-suggested LLT refinement was inadequate in 24% of overall records and increased to 38% among subjects on primary prevention and with misclassified cardiovascular risk.

**Conclusions:**

This survey highlights the need to improve the physicians’ misperceived cardiovascular risk and therapeutic inertia in patients with diabetes to successfully implement guidelines recommendations into everyday clinical practice.

**Supplementary Information:**

The online version contains supplementary material available at 10.1186/s12933-022-01495-8.

## Background

Low achievement of LDL-target is a well-established unmet clinical need with important clinical consequences among subjects with and without diabetes [1–10]. For instance, in a real-world study of almost 100,000 patients with type 2 diabetes at high or very-high cardiovascular disease (CVD) risk, we recently estimated that the achievement of guidelines-recommended LDL-c targets would reduce by one third the expected major cardiovascular events (MACE) over 10 years [10]. However, despite the widely recognized causal role of LDL-c in determining atherosclerosis and MACE [11], and the availability of treatments allowing on more than 50% of reduction of LDL-c (i.e. proprotein convertase subtilisin/kexin type 9 inhibitors [PCSK9i] or high-intensity statins combined with ezetimibe), the improvement over time in achievement of LDL-c targets has been modest [5, 9, 10], leading to missed opportunity for cardiovascular prevention. The distance between guidelines and real-world observational data is commonly reported across studies from different countries and continents, and has led to the hypothesis of systematic problems leading to this unmet clinical need [1].

Culprits for this unmet need have been often sought on the patients-side (e.g. low-adherence and excessive reporting of adverse-effect, such as the nocebo effect) [7, 12–15]. However, also among those patients with relatively high adherence to lipid-lowering treatments, the proportion of patients at high- or very-high risk being treated following guidelines recommendation is excessively low [4]. These highlights the needs to better address and dissect the possible “physician-side” parts leading to low achievement of LDL-c targets, an aspect barely considered in this field [1].

For these reasons, we conducted a survey among physicians treating patients with diabetes and dyslipidemia in several third-levels clinical centers in Italy. We aimed to dissect whether and how the physician evaluation of patients could influence the achievement of LDL-c targets. Specifically, we evaluated whether the assessment of cardiovascular risk by physicians was different from that suggested by guidelines, and weather on-going lipid-lowering treatments (LLT) or the physician-based suggestion to improve it were different from guidelines recommendations.

## Methods

### Study design

This cross-sectional self-reported survey was conducted between October 2020 and March 2021, involving overall 67 specialist physicians (i.e. endocrinologist, internal medicine or geriatricians specialist) working each-one in different third level clinical centers in Italy. Each physicians reported completely anonymized record on up to 50 patients with diabetes and dyslipidemia. The survey was conducted before the partaking to a virtual course aimed to improve awareness of LDL-cholesterol achievement in patients with diabetes. Records with incomplete information LDL-c levels or on concomitant treatments were excluded, and 2844 records were analyzed.

### Variables of interest

Information on comorbidities, life-style habits and main clinical-laboratory findings were collected. Glomerular filtration rate was estimated according to MDRD equation. Diabetic kidney disease was defined by the presence of eGFR < 60 ml/min and/or by presence of microalbuminuria or macroalbuminuria. The cardiovascular risk score was stratified in moderate, high or very-high risk as specified by EAS/EASD guidelines [16, 17]. Detailed data on treatments were reported by physicians for each patient. Ezetimibe, PCSK9i and statins molecules and dosages were provided together with information with any other LLT or nutraceutics taken by each patient. Information on adherence and adverse effects were collected by physicians and not specifically derived from pharmacy claim.

The intensity of statins treatments and overall cholesterol-lowering intensity was classified accordingly to EAS/ESC guidelines [16] and as previously described elsewhere [18]. Briefly different combination of treatments were classified in the following categories according to the expected achievable reduction in LDL-c: low cholesterol-lowering intensity (i.e. allowing < 30% LDL-c reduction, e.g. simvastatin 10 mg), moderate cholesterol-lowering intensity (i.e. allowing between 30 to 49% LDL-c reduction, e.g. rosuvastatin 5–10 mg/day or atorvastatin 10–20 mg/day or combinations such as simvastatin 10 mg + ezetimibe 10 mg), high cholesterol-lowering intensity (i.e. allowing between 50 to 59% LDL-c reduction, e.g. rosuvastatin 20–40 mg/day or atorvastatin 40–80 mg/day or combination rosuvastatin 5/10 mg + ezetimibe 10 mg), very-high intensity (i.e. allowing on average between 60 to 79% LDL-c reduction, e.g. [rosuvastatin 20–40 mg/day or atorvastatin 40–80 mg/day] plus ezetimibe 10 mg, or moderate statins with PCSK9i), and extreme intensity LLT (i.e. allowing at least 80% of LDL-c reduction, e.g. any combination of high intensity statins plus PCSK9i). Since guidelines suggest at least 50% LDL-c reduction for subjects at high or very-high CV risk, we further create a variable for LLT intensity, grouping together all treatments allowing at least 50% (i.e. high-, very-high- and extreme-intensity).

### Outcomes

#### Misclassification of CVD risk

Physicians were requested to express the cardiovascular risk assessment with the LDL-c targets deemed appropriate for each patient based on the available information. This was compared to the risk assessment and LDL-c targets recommended by EAS/ESC guidelines [16]. Each record was considered to have a “physician-based well-identified CVD risk” when the physician-based evaluation was concordant with the guideline recommendation, otherwise it was considered as “physician-based misperceived CVD risk”. Subjects without physician based assessment (n = 105) were not included in this analysis.

#### Adequate/inadequate refinement of LLT suggested by physicians

Physicians were asked to express whether and how they would have improved the LLT for each patient, and these were compared to the LLT recommended by guidelines. We defined the LLT recommended by guidelines as the LLT that would have been necessary to achieve guidelines recommended LDL-c targets in each patient, we took into account the current treatment (if any), the CVD risk of each patients, and the current distance to LDL-c targets. We then estimated the required LDL-c reduction needed to achieve the targets and we identify the “required treatment” as the combination of LLT allowing to achieve the guidelines recommended LDL-c levels (for further detail see previous publication [10]). An appropriate physician-suggested refinement of LLT was considered when it overlapped with that recommended by guidelines, or when the physician-suggested refinement had at least one-level increase in intensity compared to current treatments and was at least in the high-intensity LLT. Records of patients already reported to be at LDL-c targets were not included in these analyses.

#### Achievements of LDL-c targets

Achievement of EAS/ESC guidelines lipid-lowering targets [16, 17] were evaluated including both the absolute LDL-cholesterol thresholds (e.g. 55 mg/dl, 70 mg/dl and 100 mg/dl for patients at very-high, high and moderate CVD risk, respectively) together with the required 50% reduction from baseline LDL-c levels for patients at high or very-high CV risk. Given the cross-sectional design of the study, the baseline LDL-c levels (and the relative reduction from it) were backward estimated accordingly to the expected LDL-c reduction from the current treatment (as previously described [10, 18]). To account for possible variability in drug response, records of subjects treated with moderate lowering-intensity (allowing usually between 30–49% of reduction) were considered on target if their LDL-c was lower than the absolute LDL-c target threshold.

### Statistical analyses

Continuous variables are reported as mean and standard error, while categorical variables are reported as percentage. Given the non-complete independence of observation (data were collected by 67 different physicians from different clinical centers, each reporting data on up to 50 patients [median 47, IQR 42–49]), we evaluated the differences in clinical characteristics of patients between groups according to a generalized estimating equation (GEE) model (proc GLIMMX in SAS). This approach allowed accounting for correlated observations within each care physician. We conducted multivariable analyses (MVA) including age, sex and CV risk class as covariates, since MVA requires dataset with no missing data, and age was missing in 2% of subjects, we imputed the missing data according to the median value of the population to perform the analyses on the entire dataset. A 2-tail p-value < 0.05 was considered statistically significant. Statistical analyses were performed using SAS version 9.4 (TS1M4), graphs were produced with GraphPad Prism ver. 8

## Results

### Overall clinical characteristics

The survey collected data on 2844 patients, mostly with type 2 diabetes (94.3%) and with diabetes duration of 10 (IQR 5–16) years. As reported in Additional file 1: Table S1, around two third of the patients were at very high CVD risk (including 763 subjects with a known prior cardiovascular event), and the other third were almost entirely at high CVD risk, with only 1% of subjects (n = 31) being on moderate CVD risk. Almost half of the patients (43%) had diabetic kidney disease (defined by eGFR < 60 ml/min and/or microalbuminuria). Overall, 34% of subjects were obese and 77% with hypertension. One fifth of patients were active smokers (21%), and only 573 (20%) followed regularly a healthy diet. Regular physical activities were reported in 44% of patients (on average 2 times per week, at least 30 min of activities). Mean Hba1c was 7.3% (± 1.1) and only 4% of patients were treated with diet alone, while 71% were on metformin and 22% on DPP4i. The overall use of cardioprotective anti-hyperglycemic treatment showed a 19% of subjects being on GLP1RAs and 20% on SGLT2i.

### Lipid-lowering treatments and target achievements

**Fig. 1:**
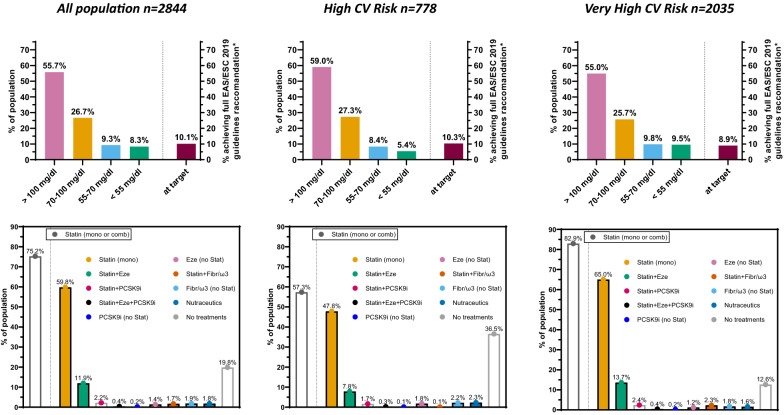
LDL-cholesterol distribution (top panels) and Lipid-lowering-Treatments (bottom panels) in the overall population and stratified by cardiovascular disease risk categories

Mean LDL cholesterol was 107 mg/dl with more than 50% of patients having LDL-c above 100 mg/dl (Fig. 1 and Additional file 1: Table S2). Overall, 20% of patients were not on active LLT. Statins were the most prescribed treatments (75%), followed by ezetimibe (14%) and PCSK9i (2.8%), nutraceutics were prescribed in 1.8% of patients. Combination of treatments allowing a high intensity LDL-c reduction (between 50 to 59% on average) were prescribed in 18%, while very-high intensity (e.g., HIS + ezetimibe) were prescribed in only 4% of patients and those on extreme-intensity (i.e. with PCSK9i and HIS) were only 7 (0.2%). As expected, subjects with very-high CVD risk were more frequently treated with higher LDL-c lowering intensity LLT (Additional file 1: Table S2). Physicians reported low adherence to LLT in 20% of the subjects, a proportion being similar across CVD risk categories. Adverse effects were reported in 7% of subjects and more frequently in those with very-high CVD risk. LLT was initiated by diabetologists, cardiologists, and primary care physicians (PCP) in 45%, 29%, and 26% of patients, respectively. Subjects with very-high CVD risk had a first LLT prescription more frequently made by a cardiologist, than by a diabetologist or a PCP. As shown in Fig. 1, 286 patients (10.1%) achieved guidelines recommended LDL-c targets (including both LDL-c levels and intensity of lipid-lowering treatments), and the median distance between mean LDL-c levels and the targets in those at high and very-high risk was 38 mg/dl (IQR 14–60 mg/dl) and 51 mg/dl (IQR 22–76 mg/dl).

### Association of misperceived CV risk with guidelines’ target achievement

**Fig. 2:**
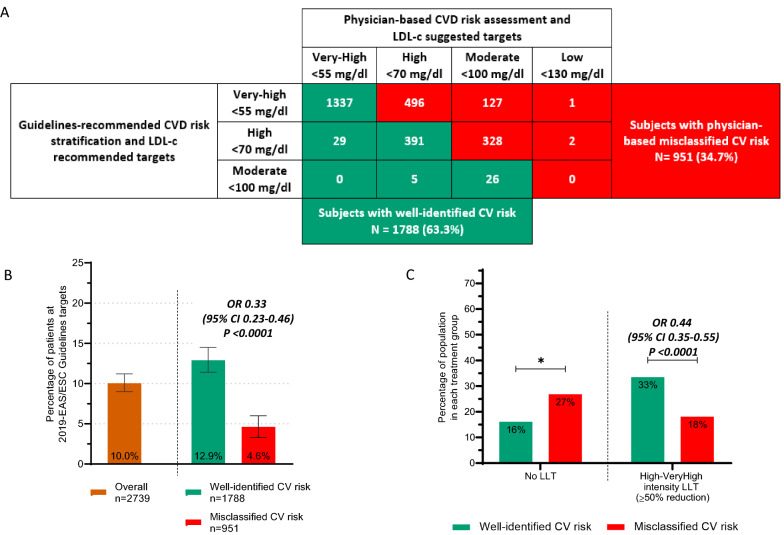
Distribution of subjects according to physician-based cardiovascular (CVD) risk assessment and LDL-c suggested targets vs. those recommended by guidelines ( **A**), and relationship of physician-based misclassified CVD risk and achievement of LDL-c targets (**B**) or current lipid-lowering treatments (LLT) (**C**). ***A***: green boxes show the number of subjects with physician-suggested LDL-c targets being equal or lower to those recommended by guidelines; Red boxes show the number of subjects with physician-suggested targets being higher than guidelines-recommended target. Patients with missing information on Physician-suggested targets (n = 105) were not included in the analysis. *Notes on ****B**** and ****C***: association between misclassified risk and achievement of LDL-c targets (B) or high-/very-high intensity lipid-lowering treatment (LLT) expressed as odds ratios, with OR < 1 suggestive of lower probability of subjects with physician-based misperceived CVD risk of achieving LDL-c targets or of being treated high/very-high- intensity LLT

When we compared the physician-based cardiovascular risk assessment and LDL-c targets to those recommended by EAS/ESC guidelines we found that only in two third of the population (n = 1754, 64%) these targets were concordant (i.e. the green boxes in Fig. 2, A). Conversely, in 951 subjects (35%) the physician wrongly identified CV risk suggesting LDL-c targets that were higher than those recommended by guidelines (i.e. the red boxes in Fig. 2, A). Subjects with misclassified CVD risk assessment had a 67% lower probability of achieving LDL-c targets as compared to those with well-classified risk (OR 0.33; 95% CI 0.23–0.46, Fig. 2 B). In line with this, as shown in Fig. 2 C, subjects with misclassified risk were more frequently untreated as compared to those with well-identified risk (27% vs 16%, respectively, p < 0.0001) and, among those on LLT, they were less frequently on high- or very-high intensive LLT (13% vs 28%, respectively with OR 0.42 [95% CI 0.33–0.53] p < 0.0001). Results were similar when the population was stratified according to CVD risk categories (Additional file 1: Figure S1).

### Factor associated with misperceived risk, LLT intensity and guidelines’ target achievement

We evaluated among subjects at high- or very-high CVD risk (i.e. 99% of the included population) which clinical factors were associated with misperceived CV-risk and whether the same was associated with the intensity of ongoing LLT and achievement of LDL-c targets. As shown in Table 1, the misperceived (underestimated) CVD risk was more likely among female subjects (OR 1.55, p = 0.001) that had also a lower probability of being treated with high-intensity treatments as compared to males (OR 0.74, p = 0.02), and a non-significant trend for the lower achievement of LDL-c targets (OR 0.87, 95% CI 0.64–1.18, p = 0.37). Independently from age and sex, the presence of prior CVD event reduced by 88% the probability of physicians’ misperceived CVD risk assessment (OR 0.12, 95% CI 0.06–0.21, p < 0.0001) and increased the probability of receiving high- or very-high intensity LLT (OR 4.02, 95% CI 3.17–5.08, p < 0.0001). This lead to a 71% higher odds of achieving LDL-c targets among those on secondary prevention compared to those on primary prevention (OR 1.71, 95% CI 1.27–2.32, p = 0.001). On the other side, the presence of other important markers of increased CVD risk (i.e. presence of arterial peripheral disease or signs of organ damages), while reducing the risk of misperceived CV risk assessment, were not associated (after adjustment for prior CVD) with higher use of intense-LLT and neither with higher achievement of LDL-c target (all p > 0.05).

**Table 1:** Clinical characteristics associated with misperceived risk, use of LLT with at least high intensity lipid-lowering-treatment LLT (allowing at least 50% of LDL-c reduction) and achievement of guidelines-recommended LDL-c targets

Characteristics	Mean ± SD or N (%)	Odds ratio of misclassified CV risk	P*	Odds ratio of being on high-intensity LLT	P*	Odds ratio of being at LDLc targets	P*
Age (ea. 5 years)	65.5 ± 10.6	**0.92 (0.86–0.99)**	**0.021**	1.03 (0.97–1.09)	0.307	**1.09 (1.00–1.17)**	**0.038**
Sex (female)	1082 (40.0%)	**1.55 (1.21–1.98)**	**0.0004**	**0.74 (0.60–0.91)**	**0.005**	0.87 (0.64–1.18)	0.374
Type 2 diabetes	2560 (94.5%)	1.38 (0.75–2.54)	0.304	1.01 (0.64–1.58)	0.975	1.73 (0.66–4.54)	0.268
Diabetes duration	11.6 ± 8.6	**0.97 (0.95–0.99)**	**0.004**	1.01 (1.00–1.02)	0.144	1.01 (1.00–1.03)	0.052
Comorbidities							
Prior CVD events	732 (27.0%)	**0.12 (0.06–0.21)**	** < 0.0001**	**4.02 (3.17–5.08)**	** < 0.0001**	**1.71 (1.27–2.32)**	**0.001**
Stroke	153 (5.6%)	1.01 (0.62–1.66)	0.967	0.45 (0.29–0.69)	0.000	1.22 (0.77–1.94)	0.402
MI	609 (22.5%)	1.59 (0.70–3.62)	0.272	2.11 (1.28–3.47)	0.003	0.78 (0.48–1.26)	0.308
Angina	102 (3.8%)	0.14 (0.05–0.36)	< 0.0001	2.76 (1.76–4.33)	< 0.0001	1.28 (0.58–2.81)	0.540
PAD	156 (5.8%)	**0.24 (0.10–0.55)**	**0.001**	1.16 (0.72–1.86)	0.546	0.75 (0.43–1.30)	0.304
Targ. organ damage	298 (11.0%)	**0.21 (0.12–0.36)**	** < 0.0001**	1.17 (0.88–1.57)	0.276	1.10 (0.75–1.59)	0.633
3 + VD risk factors	773 (28.5%)	**0.42 (0.26–0.67)**	**0.0003**	**1.45 (1.15–1.82)**	**0.002**	0.86 (0.57–1.31)	0.487
DKD	1178 (43.5%)	1.15 (0.75–1.76)	0.523	1.04 (0.81–1.32)	0.776	0.93 (0.70–1.24)	0.613
Albuminuria	796 (30.3%)	1.40 (0.87–2.25)	0.162	1.07 (0.82–1.41)	0.608	0.87 (0.62–1.24)	0.449
CKD IV stage	46 (1.8%)	1.01 (0.48–2.10)	0.983	1.52 (0.85–2.73)	0.160	2.06 (0.85–5.02)	0.111
Obesity	930 (34.3%)	0.98 (0.69–1.39)	0.903	1.18 (0.93–1.51)	0.178	**0.74 (0.58–0.94)**	**0.013**
Hypertension	2110 (77.9%)	**0.55 (0.35–0.86)**	**0.009**	1.65 (1.22–2.23)	0.001	1.07 (0.75–1.53)	0.708
COPD	221 (8.2%)	0.92 (0.62–1.37)	0.677	0.72 (0.41–1.28)	0.263	0.71 (0.36–1.41)	0.326
Life-style							
Smoke habits							
Non smokers	1546 (57.1%)	**ref.**	**0.005**	1.00 (1.00–1.00)	0.273	1.00 (1.00–1.00)	0.276
Active smoker	554 (20.5%)	**0.87 (0.64–1.17)**		1.20 (0.91–1.57)		0.72 (0.47–1.11)	
Prior smoker	608 (22.5%)	**0.59 (0.43–0.81)**		1.18 (0.92–1.52)		1.04 (0.74–1.47)	
Reg. alcohol intake	835 (31.2%)	0.85 (0.64–1.13)	0.268	1.17 (0.90–1.51)	0.234	**0.58 (0.40–0.84)**	**0.004**
Healthy diet							
No	490 (18.4%)	**ref.**	**0.010**	ref.	0.297	**ref.**	**< 0.0001**
Occasionally	1629 (61.2%)	**1.59 (1.14–2.21)**		1.10 (0.83–1.45)		**1.90 (1.20–3.02)**	
Regularly	543 (20.4%)	**1.92 (1.31–2.82)**		1.16 (0.83–1.62)		**3.09 (1.86–5.13)**	
Regular physical activity	1157 (43.7%)	1.26 (0.92–1.74)	0.151	1.09 (0.86–1.37)	0.491	1.20 (0.86–1.67)	0.296
Clinical-laboratory findings							
BMI (kg/m^2^)	29.2 ± 6.0	0.99 (0.97–1.02)	0.661	1.01 (0.99–1.03)	0.250	1.01 (0.98–1.04)	0.639
Waist (cm)	101.7 ± 14.3	0.99 (0.98–1.01)	0.323	1.01 (1.00–1.02)	0.239	1.00 (0.99–1.01)	0.839
Systolic BP (mmHg)	133.0 ± 18.1	0.99 (0.98–1.00)	0.093	1.00 (0.99–1.01)	0.885	0.99 (0.98–1.00)	0.028
Diastolic BP (mmHg)	81.4 ± 12.7	1.00 (0.99–1.02)	0.649	1.00 (0.99–1.01)	0.800	0.97 (0.96–0.99)	0.001
eGFR (ml/min)	1.0 ± 0.3	1.01 (1.00–1.01)	0.170	1.00 (0.99–1.00)	0.048	1.00 (0.99–1.00)	0.219
FPG (mg/dl)	76.0 ± 25.0	1.00 (0.99–1.00)	0.042	1.00 (1.00–1.00)	0.210	1.00 (0.99–1.00)	0.078
Hba1c (%)	139.9 ± 37.4	0.90 (0.80–1.02)	0.109	1.07 (0.98–1.16)	0.118	0.88 (0.79–0.99)	0.034
HDL-c (mg/dl)	47.1 ± 12.4	1.01 (1.00–1.02)	0.146	0.99 (0.98–1.00)	0.146	0.99 (0.98–1.00)	0.182
Triglycerides (mg/dl)	153.5 ± 67.5	1.00 (1.00–1.00)	0.597	1.00 (1.00–1.00)	0.550	1.00 (0.99–1.00)	0.067
Antidiabetic treatments							
Diet alone	90 (3.3%)	2.34 (1.43–3.82)	0.001	0.59 (0.22–1.59)	0.296	0.78 (0.35–1.76)	0.552
Metformin	1916 (70.8%)	1.03 (0.78–1.37)	0.813	1.01 (0.81–1.26)	0.920	1.07 (0.81–1.42)	0.620
Sulphonylureas	217 (8.0%)	0.86 (0.55–1.34)	0.508	0.63 (0.42–0.96)	0.030	1.07 (0.70–1.64)	0.740
Pioglitazone	97 (3.6%)	0.84 (0.43–1.66)	0.620	0.98 (0.57–1.68)	0.942	1.34 (0.66–2.71)	0.418
DPP4i	593 (21.9%)	0.91 (0.69–1.21)	0.532	0.79 (0.59–1.05)	0.104	1.05 (0.77–1.41)	0.773
GLP1RAs	515 (19.0%)	0.52 (0.39–0.69)	< 0.0001	1.23 (0.94–1.60)	0.136	1.12 (0.82–1.52)	0.469
SGLT2i	564 (20.8%)	0.97 (0.68–1.38)	0.867	1.39 (1.07–1.81)	0.015	1.46 (1.02–2.10)	0.038
Insulin	768 (28.4%)	0.76 (0.55–1.04)	0.089	1.37 (1.12–1.68)	0.003	1.02 (0.75–1.39)	0.909
Adherence/adverse effects							
Low-adherence	383 (19.5%)	0.73 (0.52–1.02)	0.067	**1.55 (1.08–2.21)**	**0.016**	0.85 (0.29–2.48)	0.770
LLT-Adverse effects	201 (7.4%)	0.64 (0.38–1.08)	0.094	1.10 (0.73–1.67)	0.650	**0.27 (0.10–0.68)**	**0.006**
Physician starting treatments							
Diabetologist	1208 (44.6%)	Ref.	0.769	Ref.	** < 0.0001**	Ref.	** < 0.0001**
Cardiologist	784 (29.0%)	0.91 (0.67–1.24)		1.35 (1.06–1.71)		**0.60 (0.42–0.85)**	
General-physician	712 (26.3%)	1.02 (0.75–1.38)		**0.48 (0.36–0.65)**		**0.25 (0.15–0.40)**	

Odds ratio (OR) above 1 suggest higher probability of achieving each outcome while OR below 1 suggest lower probability

*MI* myocardial infarction, *PAD* peripheral artery disease, *DKD* diabetic kidney disease, *CKD IV stage* subjects with eGFR < 30 ml/min, *COPD* chronic obstructive pulmonary disease, *BMI* body mass index, *eGFR* estimated glomerular filtration rate, *FPG* fasting plasma glucose

*Analyses adjusted by age, sex and prior CVD events

Among lifestyle factors, the physician-based cardiovascular risk assessment were more frequently underestimated, among non-smokers subjects and those following a regular healthy diet. However, despite this, and despite no differences in the use of high-intensity LLT, subjects with a regular healthy diet had a twofold higher probability of achieving LDL-c targets as compared to subjects not following an healthy-diet (OR 3.09, 95% CI 1.86 to 5.13, p < 0.0001). On the other side, presence of obesity or regular alcohol intake was not associated with misclassification of cardiovascular risk by physician nor with use of intense-LLT, but were associated with lower probability of achieving LDL-c targets.

Reported low-adherence to LLT and history of LLT-related adverse effects had a non-significant trend for a lower risk of misperceived physician-based cardiovascular risk assessment (O.R. 0.73, p = 0.067 and OR 0.64, p = 0.094). Among these two factors only the presence of LLT-related adverse effects was associated with lower chances of being at LDL-c targets (OR 0.26, 95% 0.10–0.67, p = 0.005), but not reported low adherence to LLT (OR 0.85, 95% CI 0.29–2.46, p = 0.8).

Finally, patients with LLT initiated by the PCP physician had no differences in physician-based cardiovascular risk assessment as compared to subjects with treatment initiated by a diabetologist, However, they had a much lower probability of being treated with high-intensity LLT (OR 0.48, 95% CI 0.36–0.65, p < 0.001) and of achieving LDL-c targets (OR 0.25, 95% CI 0.15–0.40, p < 0.001) (Table 1).

### Appropriateness of physician-based decision in increasing intensity of Lipid-Lowering treatment

**Fig. 3:**
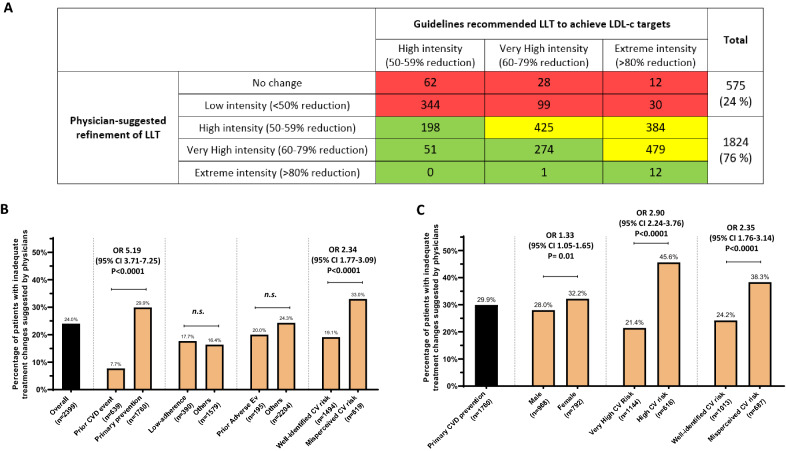
**A** physician-suggested refinement of LLT as compared to guideline-recommended refinement of LLT needed to achieve LDL-c targets. **B**, **C** Proportion of records with inadequate physicians-suggested changes in treatment, in the overall population and among those on primary prevention. **A** green boxes show the number of subjects with Physician-suggested LLT with intensity being at least equal to that recommended by guidelines. Yellow boxes show the number of subjects with physician-suggested LLT going in the same direction as that recommended by guidelines (i.e. at least one-level increase in the intensity of treatments and allowing no less than 50% LDL-c reduction). Red boxes show the number of subjects where physicians-suggested an insufficient refinement of LLT as compared to guidelines recommendation

Physicians were asked to express whether and how they would have improved the treatment for each patient included in the survey (an information collected for all except 263 subjects), and these physician suggested refinement in LLT were then compared to the guideline-recommended refinement in LLT needed to achieved the LDL-c targets (summarized on Additional file 1: Figure S1, and showing that overall high-intensity, Very-High-intensity and Extreme-intensity LLT would be required in around one third of the population each). Results of such comparison (carried out only among subjects not being at LDL-c targets nor being already on an extreme intense treatment) are shown in Fig. 3 A. We found that, as compared to EAS/ESC recommended treatments, an appropriate refinement of LLT was suggested by physicians in 76% of the records. The inadequate suggested treatment improvement (i.e. 24% of the overall population) was much higher among those on primary prevention (29.9% vs 7.7%, OR 5.19, 95% CI 3.71–7.25, p < 0.0001) while it was not affected by a history of low-adherence to LLT or LLT-related adverse effects (Fig. 3B). The inadequate suggested refinement of LLT was higher in the presence of misperceived CV risk assessment (OR 2.34, 95% CI 1.77–3.09), however, it should be noted that, also when physicians correctly identified the cardiovascular risk of patients, in 19% of cases they wrongly assigned treatment intensity (Fig. 3B). Among those without prior CVD events (i.e. ≈73% of the entire population), the inadequate decision of improving LLT was much more likely to happen among female patients (OR 1.33, 95% CI 1.05–1.65, p = 0.01), among those at high cardiovascular risk (as compared to those at very-high cardiovascular risk, OR 2.90 p < 0.0001) and among those with physician misperceived risk CV risk (OR 2.35, 95% CI 1.76–3.14, p < 0.0001, Fig. 3C).

## Discussion

Overcoming the low-achievement of guidelines recommended LDL-cholesterols targets in patients with diabetes is a worldwide major clinical challenge that is yet far from being addressed [1–10]. This survey provides novel insights through the identification of several important physician-related actionable factors that require to be improved to close the gap between guidelines recommendation and real-world clinical practice.

One of the key findings from our study is that physicians under-estimated the cardiovascular risk of patients with diabetes in around one third of cases and that such misclassification was a major determinant of low achievement of LDL-c targets. Notably, the misclassification was much higher among subjects without prior history of major cardiovascular events as compared to those on secondary prevention. Considering that in this survey 70% of those on primary prevention were at very-high CV risk (according to current guidelines classification) this suggests that physicians too often fail to consider properly those additional risk factors associated with higher cardiovascular risk. The concept of moving from a cardiovascular risk classification based simply on primary/secondary prevention towards a more comprehensive evaluation including additional risk factors (e.g. chronic kidney disease, albuminuria and obesity) to define subjects at very-high risk is relatively new but described in guidelines since almost a decade [19]. This finding suggests therefore the need to “update” the cardiovascular risk assessment by physicians. On the same line, we found that female patients were significantly more often receiving underestimated cardiovascular risk assessments as compared to male patients, and this was confirmed after adjustment for differences in age and prior history of cardiovascular disease. Under-use of LLT treatments among female has been previously reported in several studies [10, 12, 20], and while this is likely the result of several factors, our data suggest that an important one resides in the more frequently underestimated cardiovascular risk assessment by physicians in female. In line with these findings, recent studies suggested that female sex might be associated with misperceived cardiovascular assessment and treatment also among patients hospitalized with acute myocardial infarction [21, 22].

Another important finding pertains the identification of factors associated with therapeutic inertia (i.e. the lower probability of being treated with LLT having higher LDL-c lowering intensity). We found that the presence of peripheral artery disease and/or of target organ damages (i.e. albuminuria, retinopathy or left ventricular hypertension), was associated with a higher probability of receiving a correct cardiovascular risk assessment (i.e. very-high cardiovascular risk), but not of receiving higher intensity LLT (as suggested by guidelines were all these subject should be treated with LLT allowing at least a 50% reduction in LDL-c). This finding suggests that these conditions are still not perceived by several physicians as requiring more intense treatments, as opposed for example to prior history of MACE, that was instead associated with better CV risk assessment, higher use of LLT with higher LDL-c lowering intensity and therefore with higher achievement of LDL-cholesterol targets. In the same context, we found that patients initially treated by primary-care-physicians and then evaluated by a specialist had a much lower probability of receiving high-intensive treatment (52% lower odds) as compared to those initially treated by a specialist physicians. These differences were not explained by differences in age, sex or previous cardiovascular events, nor by the assessment of CV risk in these patients, suggesting, also in these cases, a possible therapeutic-inertia that ended-up in significantly lower probability of achieving LDL-c targets in these patients (75% lower odds). Therefore, while the reasons for such resilience are unclear (it might be derived both from patients’ and physicians’ standpoints) it requires to be improved. Given these findings and the increase in the availability of different LLT options, it becomes essential to tackle therapeutic-inertia on LLT in a similar fashion to what has been advocated by ADA/EASD guidelines for management of glucose-lowering treatments [23].

One of the well-reported and documented factors influencing the achievement of LDL-c targets is the adherence to the prescribed LLT [7, 12–14, 24]. In this context, we found that the adherence to treatment as reported by the physician (without any data such as pharmacy claims or collection) was instead only marginally and not significantly associated with achievements of LDL-c targets. Given the intrinsic relationship between adherence and LDL-c reduction, such data might suggest that “physician-perceived” low adherence to treatment is likely an ineffective measure of true adherence to treatments. Although recent studies reported a slight improvement in adherence to LLT over time [5], and that the low achievement of LDL-c target is present also among subjects with relatively high adherence to treatments [4], having a proper measure of adherence is essential both for physicians and patients to understand difficulties in achieving targets. Therefore, these data advocate the importance of providing better tools, e.g. using integrated information from pharmacy claims to help patients and physicians effectively measuring adherence (i.e. measuring outpatient medication dispensing). On the other side, adverse effects to LLT are another aspects known to impede adherence and the use of more intense LLT, and therefore to reduce the achievement of LDL-c targets, as confirmed in this survey [7, 12–14]. However, the presence of adverse events in this population was found only in about 8% of records, in line with other real-world studies [25, 26]. Therefore, although these numbers are higher than those reported in RCTs [15, 27], it remains a relatively low prevalence, which could explain only in a minor part the overall low rate of achievements of LDL-c targets in real-world studies.

Finally, this study allowed to evaluate whether physicians suggested treatment improvement in LLT in line with those recommended by guidelines to achieve LDL-c targets. First, we found that, following guidelines recommendation, the proportion of subjects needing high-intensity LLT, very-high-intensity LLT, and extreme intensity LLT (i.e. allowing 50 to 59%, 60% to 79% and more than 80% LDL-c reduction, respectively) in this population of subjects at high or very-high CV risk with diabetes were, 35%, 32% and 33%, respectively (in line with previous reports on larger population with diabetes [4, 10]). Interestingly only 22% of patients requiring LLT intensification had a perfect overlap between physician-suggested LLT and guidelines-recommended LLT (i.e. the green boxes in Fig. 3A). However, in other 54% of patients the physician-suggested treatment improvement was in the “correct direction” (i.e. at least one-step increase in intensity and with no less than 50% LDL-c reduction). Conversely, in the remaining 24% of cases physicians did not recommend changes in LLT or recommended changes with the insufficient LDL-c lowering intensity. Surprisingly, we did not find an influence of adverse-event or low-adherence on the proportion of subjects receiving an inadequate physicians-proposed change in treatment, suggesting therefore that hesitancy to consider a more effective treatment is not justified by a history of adverse effects or low-adherence. On the other hand, we found that misclassification of cardiovascular risk was strongly associated with an inadequate decision on LLT improvement. This was strikingly evident among those on primary prevention, where almost 1 out of 2 patients had inadequate physician-suggested refinement of LLT. Aligning physician-based decision on LLT intensification to the guidelines recommendation in these subjects appears therefore extremely important, also because most of these subjects are at very-high cardiovascular risk (as reported also in other real-world studies on type 2 diabetes [28]).

Beyond providing novel insights into the problem of low achievement of LDL-c targets, this study also highlights the complexity of the process leading to physicians’ perception of cardiovascular risk and how this is translated (or not) into active and appropriate clinical actions (i.e. prescription of adequate treatment). For instance, our study suggests the presence of at least two different scenarios. In the first one, physicians correctly identified the cardiovascular risk and LDL-c targets to be achieved by their patients (i.e. around two third of the cases) but then this is not translated into adequate treatment intensity (in 19% of cases in this study). In the second scenario, physicians underestimate the cardiovascular risk or do not identify the appropriate LDL-c targets, and therefore under-treat and under-control their patients. Further studies will be needed to identify the complex causes of these different scenarios leading to therapeutic inertia (e.g. presence of health care system-related barriers, costs, fear of adverse events, etc.) and to identify new ways to help physicians to properly asses the cardiovascular risk of patients (e.g. providing more time to visit patients and/or providing workshop/courses to physicians on cardiovascular prevention) and to help them taking a step forward to achieve the therapeutic goals (e.g. discuss the value of maximal LDL-c lowering treatment, the advantages of using combined LLT, and to utilize regular monitoring for efficacy and adherence).

Some limitations of our study must be acknowledged. First this is a cross-sectional survey therefore it can only suggest association and further studies are required to assess causality (e.g. prospective studies, ideally with randomized intervention, testing whether improving physician CV risk assessment influences LLT prescription and the achievement of LDL-c targets). Second, we used complete anonymized data collected and reported by physicians, and we could not exclude bias due to the self-reporting design of the study (i.e. under the impression of being evaluated, physicians could have unawarely reported data differently from what would have happened in real-clinical practice). However, such bias generally goes towards the direction of reporting treatments and recommendations being more similar to those expected from guidelines, therefore if present, such biases would have pushed the results towards the null hypothesis of no association between misperceived CV risk or inadequate improvement of LLT and LDL-c target achievements. Third, as expected, some variable had missing information, e.g. adherence was missing in 20% of cases, and such information might be not missing at random (i.e. those with lower adherence are more likely to be those for whom the information was not collected). While important, this aspect reinforce the need to provide physicians with tolls providing reliable information on adherence (e.g. integration with pharmacy claims). Finally, from a generalizability standpoint, it must be considered that the interviewed physician were all from Italy, and although national and international guidelines for dyslipidemia and cardiovascular management in diabetes are highly overlapping across countries and continents [17, 23, 29], clinical practice is influenced by country and region-specific factors (e.g. nationals health systems and accessibility to drugs), and these results might not be applicable in other settings. However, this limitation highlights the need for additional studies, like this one, also using data from other countries.

Beyond limitations, the main strengths of this manuscript reside in the detailed information collected from a physician-based perspective, including perceived cardiovascular risk assessment and suggested intensification of LLT. This allowed the identification of actionable items that might improve LLT management in the real-world setting targeting the improvement of physician-based decisions. Indirect evidence supporting the possible benefit of such an approach is provided by a recent study were a “pay-for-performance model” showed a modest but significant increase in the correct use of cardiovascular preventive medications (including LLT) across different organizations in the united states [30]. Therefore, while future innovation should go towards a better definition of cardiovascular risk (e.g. using genetic data) [31, 32] and the precise definition of subjects who can take the most advantage from more intensive treatments [10, 33, 34], these needs go together with the one urgently advocating the correct application of current knowledge and therapeutic armamentarium. As previously estimated, implementing dyslipidemia guidelines into every day clinical practice would have a major impact on reduction of the occurrence of future cardiovascular events in patients with diabetes [1, 10].

## Conclusions

In conclusion, through the analyses of the physician-side perspective in the management of patients with diabetes and dyslipidemia, we identify several actionable items needing to be addressed to increase the achievement of LDL-c targets. While our findings require further validations and advocate for similar studies in other settings, they highlight how the improvement of cardiovascular risk-assessments by physicians and of physician-decision on intensification of LLT (e.g. through teaching workshop) will be essential points to be addressed to successfully implement dyslipidemia guidelines into everyday clinical practice, and reduce the cardiovascular burden in patients with diabetes.

## Supplementary Information

**Additional file 1:** **Table S1.** Clinical characteristics in the overall population and stratified by high or very high CV risk. **Table S2.** Lipid lowering treatments, adherence and adverse events in the overall population and stratified by high or very high CV risk. **Figure S1.** Relationship of physician-based misclassified CVD risk and achievement of LDL-c targets or current lipid-lowering treatments. **Figure S2.** Guidelines-recommended treatments that would be necessary to achieve guidelines recommended LDL-c targets in the described population.

## Data Availability

The data that support the findings of this study are available from the corresponding author upon reasonable request.

## Abbreviations

CHD: Coronary heart disease
CVD: Cardiovascular disease
EAS: European Atherosclerosis Society
EASD: European Association for the Study of Diabetes
eGFR: Estimated glomerular filtration rate
ESC: European Society of Cardiology
GEE: Generalized estimating equation
HDL: High density lipoprotein
HIS: High-intensity-statin
LDL: Low dense lipoprotein
LLT: Lipid lowering treatment
MACE: Major adverse cardiovascular events
MDRD: Modification of diet in renal disease study equation
MVA: Multivariable adjusted models
O.R.: Odds ratio
PCSK9i: Proprotein convertase subtilisin/kexin type 9 inhibitors
