# Correction to: Physicians’ misperceived cardiovascular risk and therapeutic inertia as determinants of low LDL-cholesterol targets achievement in diabetes

**DOI:** 10.1186/s12933-022-01515-7

**Published:** 2022-05-13

**Authors:** Mario Luca Morieri, Olga Lamacchia, Enzo Manzato, Andrea Giaccari, Angelo Avogaro, Lipid-Lowering-Relevance Study Group, Lucio Amoresano, Stefania Angotti, Laura Bartone, Francesco Caraffa, Antonello Carboni, Stefano Carro, Silvestre Cervone, Alessandra Clerico, Ida Console, Danilo Mario Conti, Sergio D’Addato, Alessandra de Bellis, Francesco de Meo, Alberto di Carlo, Graziano di Cianni, Giuseppe di Giovanni, Sergio di Lembo, Fabrizio Diacono, Mara Dolcino, Giovanni Elia, Paolo Elli, Cristina Fatone, Angelica Galli, Giovanni Galluzzo, Adriana Garzaniti, Renata Ghelardi, Anna Giacchini, Loretta Giunta, Francesco Golia, Franco Gregorio, Dario Ierna, Antonio Lampitella, Antonio Luciano, Ada Maffettone, Raffaele Mancini, Ida Mangone, Linneo Enzo Mantovani, Alberto Marangoni, Giuseppe Marelli, Narciso Marin, Gennaro Marino, Eugenio Mastromatteo, Gaetano Mazziotti, Elisa Me, Giuseppe Memoli, Laura Silvia Maria Menicatti, Simona Moffa, Manuela Moise’, Fabrizio Monaco, Sara Nazzarena Morgante, Francesca Pellicano, Ettore Petraroli, Deamaria Piersanti, Antonino Pipitone, Susanna Puglisi, Maura Rinaldi, Mario Rizzo, Maura Rosco, Giampaolo Scollo, Natalino Simioni, Mariarosaria Squadrone, Giacomo Sturniolo, Anna Tedeschi, Biagio Tizio, Diletta Ugolotti, Livio Valente, Carmela Vinci, Luca Zenoni, Maria Grazia Zenti

**Affiliations:** 1 Department of Medicine, University of Padova, via Giustiniani 2 IT, 35128 Padova, Padua Italy; 2 Department of Medical and Surgical Sciences, University of Foggia, Foggia, Italy; 3 Center for Endocrine and Metabolic Diseases, Department of Surgical and Medical Sciences, Fondazione Policlinico Universitario A. Gemelli IRCCS-and Università Cattolica del Sacro Cuore, Rome , Italy

## Correction to: Cardiovascular Diabetology (2022) 21:57https://doi.org/10.1186/s12933-022-01495-8

Following publication of the original article [1], the authors identified an error in the author name of Angelo Avogaro.

- The incorrect author name is: Angelo Avogardo
- The correct author name is: Angelo Avogaro

Also, the word “Diabetes Unit” should be removed from the first affiliation for the authors, “Mario Luca Morieri, Enzo Manzato and Angelo Avogaro”.

The author group has been updated above and the original article has been corrected.
