# Supplementary material for: Physicians’ misperceived cardiovascular risk and therapeutic inertia as determinants of low LDL-cholesterol targets achievement in diabetes
Source: Cardiovasc Diabetol. 2022 Apr 26;21:57. doi: 10.1186/s12933-022-01495-8 (PMC9044595; doi:10.1186/s12933-022-01495-8)
Supplement: Supplementary file 1 — Additional file 1: Table S1. Clinical characteristics in the overall population and stratified by high or very high CV risk. Table S2. Lipid lowering treatments, adherence and adverse events in the overall population and stratified by high or very high CV risk. Figure S1. Relationship of physician-based misclassified CVD risk and achievement of LDL-c targets or current lipid-lowering treatments. Figure S2. Guidelines-recommended treatments that would be necessary to achieve guidelines recommended LDL-c targets in the described population. [file 12933_2022_1495_MOESM1_ESM.docx]

List of Additional file 1 materials of paper by Morieri et al. “***Physicians’ misperceived cardiovascular risk and therapeutic inertia as determinants of low LDL-cholesterol targets achievement in diabetes”***

1. **Acknowledgments**
2. **Additional file 1 Tables S1 and S2**
3. **Additional file 1 Figures S1 and S2**
4. **Acknowledgments:** The Authors acknowledge all the members of the Lipid Lowering Relevance Study group: “LUCIO AMORESANO - Department of Endocrinology, ASST Valtellina e Alto Lario, Sondrio; STEFANIA ANGOTTI - Diabetes Unit, ACISMOM, Roma; LAURA BARTONE - Department of Diabetology, Hospital Gardone Val Trompia, ASST Spedali Civili di Brescia ; FRANCESCO CARAFFA - Department of Diabetology, ASL TO 4 Hospital of Ciriè e Lanzo T.se, Torino; ANTONELLO CARBONI - Department of Diabetology, ASSL Sassari; STEFANO CARRO - Department of Diabetology, ASL5 Spezzino; SILVESTRE CERVONE - Diabetes Unit, DSS 52 ASL Foggia; ALESSANDRA CLERICO - Department of Diabetology, ASL Città di Torino; IDA CONSOLE - Diabetes Unit, ASL Bari; DANILO MARIO CONTI - Diabetes Unit, ASST Lodi; SERGIO D'ADDATO - Department of Medicine, IRCSS University Hospital, Bologna; ALESSANDRA DE BELLIS - Departmnent of Diabetology, Hospital S.M.Annunziata, Bagno a Ripoli, Firenze; FRANCESCO DE MEO – Diabetology Unit of Gaeta ASL LT; ALBERTO di CARLO - Department of Diabetology, USL Toscana Nord Ovest, Lucca; GRAZIANO DI CIANNI - Depatment of Diabetology, ASL Toscana Nord Ovest, Hospital of Livorno; GIUSEPPE DI GIOVANNI - Diabetes Unit, ASL Napoli1 Centro, Napoli; SERGIO DI LEMBO - Diabetes Unit, ASST Cremona; FABRIZIO DIACONO - Diabetes Unit, ASL Lecce; MARA DOLCINO - Diabetes Unit, ASL Alessandria; GIOVANNI ELIA - Department of Medicine, Hospital Giovanni Paolo II, ASP 7, Ragusa; PAOLO ELLI - Department of Endocrinology, Hospital S. Anna ASST-Lariana, Como; CRISTINA FATONE - Diabetes Unit, USL Umbria 1; ANGELICA GALLI - Department of Diabetology, N.E. ASL Latina ; GIOVANNI GALLUZZO - Diabetes Unit, ASP Agrigento; ADRIANA GARZANITI - Diabetes Unit, ASST  Pavia; RENATA GHELARDI - Department of Diabetology, ASST Melegnano Martesana (MI); ANNA GIACCHINI - Diabetes Unit, ASL RM3, Casa della Salute di Ostia, Roma ; LORETTA GIUNTA - Department of Diabetology, AOU G. Martino, Messina; FRANCESCO GOLIA - Diabetes Unit, ASL Caserta; FRANCO GREGORIO - Department of Diabetology, Hospital of Jesi e Fabriano, Ancona; DARIO IERNA - Diabetes Unit, ASP 208, Siracusa; ANTONIO LAMPITELLA - Diabetes Unit, Aversa (CE); ANTONIO LUCIANO - Diabetes Unit, ASL Benevento; ADA MAFFETTONE - Department of Medicine, AOS Dei Colli, Napoli; RAFFAELE MANCINI - Diabetes Unit, SSD ASP Catanzaro; IDA MANGONE - Department of Medicine, Hospital of Vimercate (MB); LINNEO ENZO MANTOVANI - Diabetes Unit, Poliambulatorio Armonia, Mantova; ALBERTO MARANGONI - Department of Diabetology, ASL n. 7 Pedemontana, Bassano del Grappa, Vicenza; GIUSEPPE MARELLI - Department of Diabetology, Fatebenefratelli Hospital, Erba, Como; NARCISO MARIN - Diabetologia ULSS 2 Marca Trevigiana; GENNARO MARINO - Diabetes Unit, ASL Napoli 1 Centro , CAD2 Ponticelli , Napoli ; EUGENIO MASTROMATTEO - Diabetes Unit, ASL BAT, Spinazzola; GAETANO MAZZIOTTI - Department of Diabetology, Hospital of Crotone; ELISA ME - Diabetes Unit, ASL TO3, Torino; GIUSEPPE MEMOLI - Diabetes Unit, San Luca, Ariano Irpino, Avellino; LAURA SILVIA MARIA MENICATTI - Diabetes Unit, Istituto Clinico S. Siro, San Donato Group, Milano; SIMONA MOFFA - Diabetes Unit, Fondazione Policlinico Gemelli IRCCS, Roma; MANUELA MOISE' - Diabetes Unit, Distretto 2, ASL 3 Serenissima Venezia-Mestre; FABRIZIO MONACO - Department of Diabetology, Hospital Gaetano Bernabeo, Ortona (CH); SARA NAZZARENA MORGANTE - Department of Diabetology, Hospital San Salvatore, Aquila; FRANCESCA PELLICANO - Department of Diabetology, Hospital Santa Maria delle Croci, Ravenna, AUSL Romagna; ETTORE PETRAROLI - Diabetes Unit, Capua ASL Caserta; DEAMARIA PIERSANTI - Department od Diabetology, Hospital Santi Filippo e Nicola, Avezzano (AQ); ANTONINO PIPITONE - Department of Diabetology, Hospital of Adria, Rovigo; SUSANNA PUGLISI - Diabetes Unit, ASP Messina; MAURA RINALDI - Diabetes Unit, ASL Verbano-Cusio-Ossola; MARIO RIZZO - Department of Daibetology, Hospital Buccheri La Ferla FBF, Palermo; MAURA ROSCO - Diabetes Unit, ASL Bari; GIAMPAOLO SCOLLO - Department of Medicine, Hospital S. Anna, Como; NATALINO SIMIONI - Department of Medicine, Hospital of Cittadella, AUSSL 6 Euganea, Padova ; MARIAROSARIA SQUADRONE - Diabetes Unit, ASL Vasto (IS); GIACOMO STURNIOLO - Diabetes Unit, ASST Lariana; ANNA TEDESCHI - Department of Diabetology, Hospital San Jacopo, USLCentro Toscana, Pistoia; BIAGIO TIZIO - Diabetes Unit, DS 64-Eboli ASL Salerno; DILETTA UGOLOTTI - Diabetes Unit, AUSL Parma ; LIVIO VALENTE - Diabetes Unit, ASL Frosinone; CARMELA VINCI - Department of Diabetology, AULSS4 Veneto, San Dona di Piave, Venezia; LUCA ZENONI - Department of Medicine, Hospital of Seriare, ASST Bergamo Est; MARIA GRAZIA ZENTI - Division of Endocrinology, Diabetes and Metabolism, Department of Medicine, University Hospital of Verona

**Additional file 1: Table S1: Clinical characteristics in the overall population and stratified by high or very high CV risk.**

| Characteristics | Avail | Overall  N = 2844 | High CV risk  N=778 | Very High CV risk  N=2035 | P |
| --- | --- | --- | --- | --- | --- |
| Age (years) | 98% | 65.3 ± 11.0 | 61.8 ± 12.2 | 67.0 ± 9.6 | <0.0001 |
| female | 100% | 1137 (40.0%) | 400 (51.4%) | 726 (35.7%) | <0.0001 |
| Type 1 diabetes | 100% | 145 (5.1%) | 57 (7.3%) | 76 (3.7%) | 0.001 |
| Type 2 diabetes | 100% | 2682 (94.3%) | 717 (92.2%) | 1946 (95.6%) |  |
| Diabetes Duration (years) | 97% | 10 (5-16) | 7 (3-12) | 10 (6-18) | <0.0001 |
| *Comorbidities:* |  |  |  |  |  |
| Very High CV risk | 100% | 2035 (71.6%) | 0 (0.0%) | 2035 (100.0%) | n.a. |
| High CV risk | 100% | 778 (27.4%) | 778 (100.0%) | 0 (0.0%) | n.a. |
| Moderate CV risk | 100% | 31 (1.1%) | 0 (0.0%) | 0 (0.0%) | n.a. |
| Prior CVD events | 100% | 762 (26.8%) | 0 (0.0%) | 762 (37.4%) | n.a. |
| Stroke | 100% | 165 (5.8%) | 0 (0.0%) | 165 (8.1%) | n.a. |
| Myocardial infarction | 100% | 628 (22.1%) | 0 (0.0%) | 628 (30.9%) | n.a. |
| Angina | 100% | 102 (3.6%) | 0 (0.0%) | 102 (5.0%) | n.a. |
| PAD | 100% | 159 (5.6%) | 0 (0.0%) | 159 (7.8%) | n.a. |
| Target organ damage | 100% | 302 (10.6%) | 0 (0.0%) | 302 (14.8%) | n.a. |
| 3+ CV risk factors | 100% | 794 (27.9%) | 0 (0.0%) | 794 (39.0%) | n.a. |
| Diabetic Kidney Disease | 100% | 1228 (43.2%) | 86 (11.1%) | 1142 (56.1%) | <0.0001 |
| Albuminuria (micro or macro) | 97% | 827 (30.0%) | 0 (0.0%) | 827 (42.0%) | n.a. |
| CKD IV stage | 93% | 52 (2.0%) | 0 (0.0%) | 52 (2.7%) | n.a. |
| Obesity | 100% | 960 (33.8%) | 85 (10.9%) | 875 (43.0%) | <0.0001 |
| Hypertension | 100% | 2190 (77.0%) | 352 (45.2%) | 1838 (90.3%) | <0.0001 |
| COPD | 100% | 227 (8.0%) | 20 (2.6%) | 207 (10.2%) | <0.0001 |
| *Life-style:* |  |  |  |  |  |
| Non Smokers | 100% | 1629 (57.3%) | 601 (77.2%) | 1001 (49.2%) | <0.0001 |
| Active Smoker | 100% | 579 (20.4%) | 88 (11.3%) | 491 (24.1%) |  |
| Prior Smoker | 100% | 636 (22.4%) | 89 (11.4%) | 543 (26.7%) |  |
| Regular Alcohol consumer | 99% | 859 (30.6%) | 175 (22.7%) | 679 (33.8%) | <0.0001 |
| No healthy diet | 98% | 508 (18.2%) | 96 (12.5%) | 411 (20.6%) | <0.0001 |
| Occasional healthy diet | 98% | 1715 (61.3%) | 444 (58.0%) | 1257 (62.9%) |  |
| Regular healthy diet | 98% | 573 (20.5%) | 225 (29.4%) | 332 (16.6%) |  |
| Regular physical activities | 98% | 1233 (44.4%) | 448 (58.7%) | 758 (38.2%) | <0.0001 |
| *Clinical-laboratory findings:* |  |  |  |  |  |
| BMI kg/m2 | 96% | 29.1 ± 5.9 | 27.1 ± 5.9 | 29.9 ± 5.8 | <0.0001 |
| Waist cm | 75% | 101.7 ± 14.4 | 96.0 ± 13.7 | 103.9 ± 14.1 | <0.0001 |
| Sistolic BP mmHg | 98% | 132.9 ± 18.2 | 127.8 ± 16.5 | 135.0 ± 18.3 | <0.0001 |
| Diastolic BP mmHg | 98% | 81.3 ± 12.6 | 78.9 ± 11.1 | 82.3 ± 13.0 | <0.0001 |
| eGFR (ml/min/1.74m^2^) | 93% | 76.3 ± 25.8 | 84.5 ± 22.0 | 72.6 ± 25.4 | <0.0001 |
| fasting plasma glucose (mg/dl) | 98% | 139.7 ± 37.5 | 135.2 ± 35.6 | 141.8 ± 38.0 | 0.0017 |
| Hba1c (%) | 100% | 7.3 ± 1.1 | 7.1 ± 1.1 | 7.3 ± 1.1 | 0.0004 |
| Total Cholesterol (mg/dl) | 97% | 184.3 ± 42.7 | 187.0 ± 38.5 | 183.4 ± 44.3 | <0.0001 |
| HDL-cholesterol (mg/dl) | 96% | 47.2 ± 12.5 | 50.7 ± 12.9 | 45.7 ± 12.0 | <0.0001 |
| Triglycerides (mg/dl) | 96% | 153.1 ± 68.0 | 139.0 ± 60.8 | 159.1 ± 70.1 | <0.0001 |
| LDL-cholesterol (mg/dl) | 100% | 106.7 ± 37.1 | 108.3 ± 34.3 | 106.2 ± 38.2 | 0.0003 |
| NonHDL-cholesterol (mg/dl) | 96% | 136.9 ± 41.6 | 135.9 ± 37.8 | 137.6 ± 43.1 | 0.10 |
| *Antidiabetic treatments:* |  |  |  |  |  |
| Diet treatment | 100% | 95 (3.3%) | 54 (6.9%) | 41 (2.0%) | <0.0001 |
| Metformin | 100% | 2008 (70.6%) | 576 (74.0%) | 1414 (69.5%) | 0.07 |
| Sulphonylureas | 100% | 228 (8.0%) | 62 (8.0%) | 164 (8.1%) | 0.47 |
| Pioglitazone | 100% | 109 (3.8%) | 41 (5.3%) | 67 (3.3%) | 0.02 |
| DPP4i | 100% | 618 (21.7%) | 177 (22.8%) | 440 (21.6%) | 0.29 |
| GLP1RAs | 100% | 528 (18.6%) | 95 (12.2%) | 432 (21.2%) | <0.0001 |
| SGLT2i | 100% | 577 (20.3%) | 96 (12.3%) | 479 (23.5%) | <0.0001 |
| Insulin | 100% | 802 (28.2%) | 140 (18.0%) | 649 (31.9%) | <0.0001 |

**Additional file 1: Table S2: Lipid lowering treatments, adherence and adverse events in the overall population and stratified by high or very high CV risk.**

| Characteristics | Avail | Overall  N = 2844 | High CV risk  N=778 | Very High CV risk  N=2035 | P |
| --- | --- | --- | --- | --- | --- |
| *LDL-c lowering intensity:* |  |  |  |  |  |
| No treatments | 100% | 564 (19.8%) | 284 (36.5%) | 257 (12.6%) | <0.0001 |
| Low (<30% reduction) | 100% | 249 (8.8%) | 79 (10.2%) | 168 (8.3%) |  |
| Moderate (30-49% reduction) | 100% | 1389 (48.8%) | 327 (42.0%) | 1056 (51.9%) |  |
| High (50-59% reduction) | 100% | 512 (18.0%) | 70 (9.0%) | 442 (21.7%) |  |
| Very-high (60%-79% reduction) | 100% | 123 (4.3%) | 17 (2.2%) | 106 (5.2%) |  |
| Extreme (>80% reduction) | 100% | 7 (0.2%) | 1 (0.1%) | 6 (0.3%) |  |
| *Lipid-lowering class:* |  |  |  |  |  |
| Statins (alone or combined) | 100% | 2140 (75.2%) | 446 (57.3%) | 1688 (82.9%) | <0.0001 |
| High-intensity statins (HIS) | 100% | 345 (12.1%) | 28 (3.6%) | 317 (15.6%) | <0.0001 |
| Moderate-intensity statins | 100% | 1594 (56.0%) | 368 (47.3%) | 1220 (60.0%) | <0.0001 |
| Ezetimibe (alone or combined) | 100% | 379 (13.3%) | 75 (9.6%) | 303 (14.9%) | <0.0001 |
| Statins + Ezetimibe | 100% | 339 (11.9%) | 61 (7.8%) | 278 (13.7%) | <0.0001 |
| HIS + Ezetimibe | 100% | 57 (2.0%) | 3 (0.4%) | 54 (2.7%) | 0.0001 |
| PCSK9i | 100% | 79 (2.8%) | 16 (2.1%) | 63 (3.1%) | 0.28 |
| Statins+Eze+PCSK9i | 100% | 11 (0.4%) | 2 (0.3%) | 9 (0.4%) | 0.19 |
| PCSK9i w/o statins | 100% | 6 (0.2%) | 1 (0.1%) | 5 (0.2%) | 0.53 |
| Statins + Fibrates/Omega3 | 100% | 40 (1.4%) | 14 (1.8%) | 25 (1.2%) | 0.234 |
| Ezetimibe w/o statins | 100% | 53 (1.9%) | 17 (2.2%) | 36 (1.8%) | 0.465 |
| Fibrates/omega3 w/o statins | 100% | 52 (1.8%) | 18 (2.3%) | 33 (1.6%) | 0.003 |
| Nutraceutics | 100% | 40 (1.4%) | 14 (1.8%) | 25 (1.2%) | 0.23 |
| *Adherence and adverse effects:* |  |  |  |  |  |
| Low-adherence | 72% | 413 (20.3%) | 101 (21.5%) | 311 (19.9%) | 0.41 |
| Adverse effects | 100% | 207 (7.3%) | 46 (5.9%) | 161 (7.9%) | 0.03 |
| Myopathy | 100% | 106 (3.7%) | 23 (3.0%) | 83 (4.1%) | 0.21 |
| Liver-related | 100% | 8 (0.3%) | 0 (0.0%) | 8 (0.4%) |  |
| Intolerance to statin | 100% | 100 (3.5%) | 22 (2.8%) | 78 (3.8%) | 0.07 |
| Suspension of statin | 100% | 73 (2.6%) | 16 (2.1%) | 57 (2.8%) | 0.23 |
| *Physician starting treatments:* |  |  |  |  |  |
| Diabetologist | 100% | 1283 (45.1%) | 415 (53.3%) | 853 (41.9%) | <0.0001 |
| Cardiologist | 100% | 826 (29.0%) | 144 (18.5%) | 673 (33.1%) |  |
| General-Physician | 100% | 731 (25.7%) | 218 (28.0%) | 506 (24.9%) |  |

**Additional file 1: figure S1: Relationship of physician-based misclassified CVD risk and achievement of LDL-c targets or current lipid-lowering treatments.**


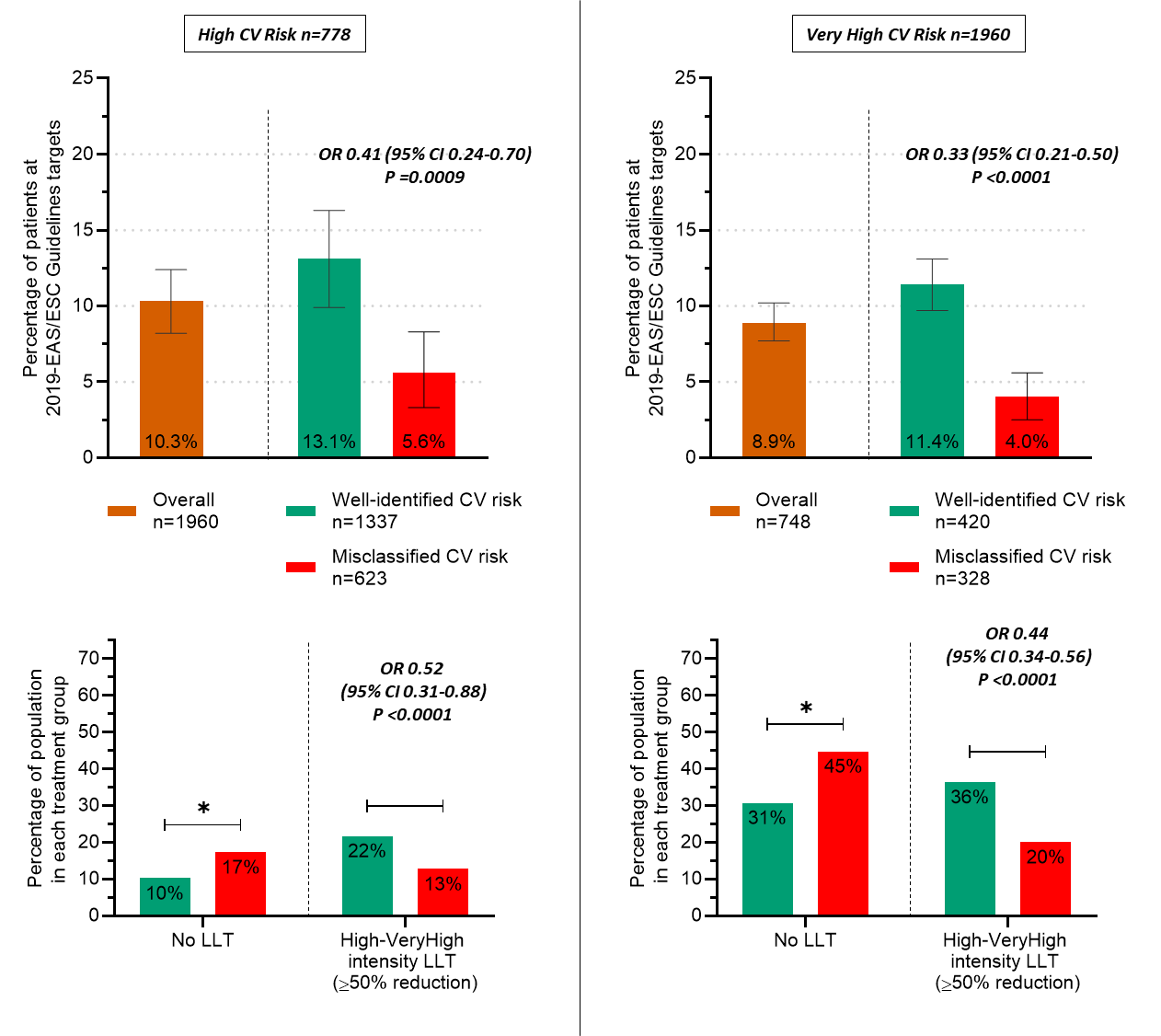


**Additional file 1: figure S2: Guidelines-recommended treatments that would be necessary to achieve guidelines recommended LDL-c targets in the described population.**

**
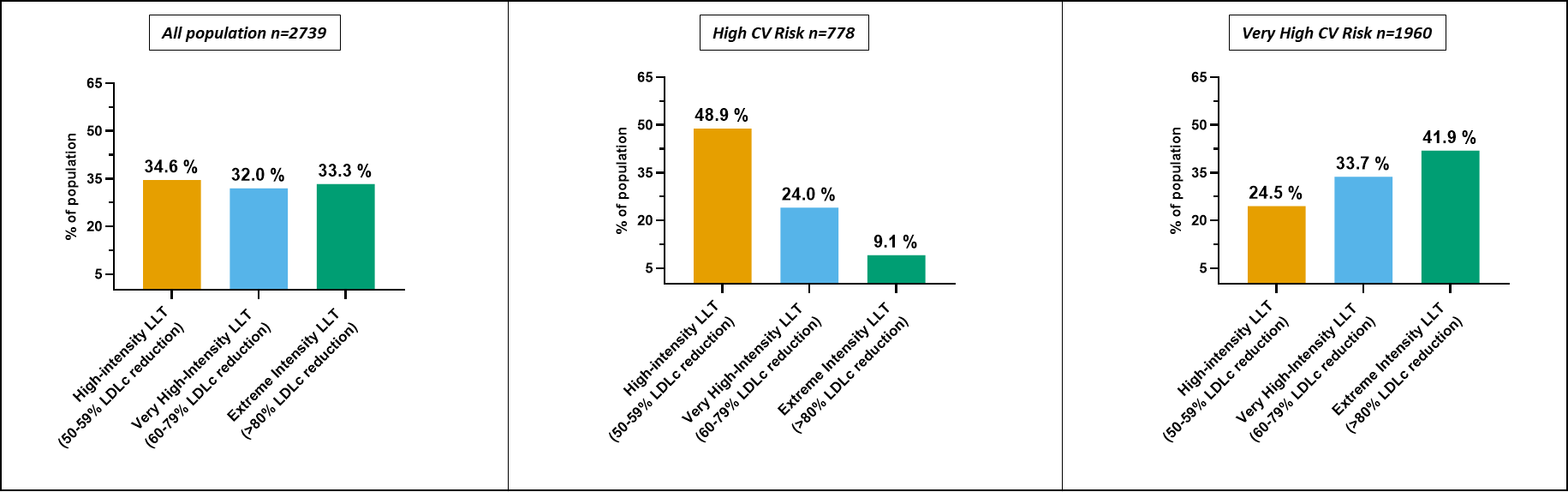
**
